# Decision making for concomitant high tibial osteotomy (HTO) in cartilage repair patients based on a nationwide cohort study of 4968 patients

**DOI:** 10.1007/s00402-020-03476-6

**Published:** 2020-05-23

**Authors:** Svea Faber, Johannes Zellner, Peter Angele, Gunter Spahn, Ingo Löer, Wolfgang Zinser, Philipp Niemeyer

**Affiliations:** 1 OCM | Orthopädische Chirurgie München, Steinerstrasse 6, 812306 München, Germany; 2 St. Joseph Krankenhaus, Regensburg, Germany; 3 Sporthopaedicum, Berlin, Straubing, Regensburg, Germany; 4 Klinik für Unfallchirurgie, Universitätsklinikum Regensburg, Regensburg, Germany; 5 Praxisklinik Eisenach, Eisenach, Germany; 6 Klinik für Unfall-, Hand- und Wiederherstellungschirurgie, Universitätsklinikum Jena, Jena, Germany; 7 Orthopädie in Essen, Essen, Germany; 8 St. Vinzenz-Hospital, Dinslaken, Germany; 9 Klinik für Orthopädie und Traumatologie, Universtitätsklinikum Freiburg, Freiburg, Germany

**Keywords:** High tibial osteotomy, Cartilage surgery, Cartilage repair, Factors, Concomitant surgery

## Abstract

**Background:**

High tibial osteotomy (HTO) for varus deformities is a common concomitant treatment in cartilage surgery. Aim of the present study was to analyze factors influencing the decision towards accompanying HTO in patients with cartilage defects of the medial femoral condyle, such as the amount of varus deformity.

**Methods:**

Data from 4986 patients treated for cartilage defects of the knee from the German Cartilage Registry (KnorpelRegister DGOU) were used for the current analysis. Seven hundred and thirty-six patients fulfilled the inclusion criteria. Their data were analyzed for factors influencing the decision towards performing a concomitant HTO using *t* test, univariate and multivariate binary logistic regression models.

**Results:**

The break point at which the majority of patients receive a concomitant HTO is 3° of varus deformity. Several factors apart from the amount of varus deformity (5.61 ± 2.73° vs. 1.72 ± 2.38°, *p* < 0.00) differed significantly between the group of patients with HTO and those without. These included defect size (441.6 ± 225.3 mm^2^ vs. 386.5 ± 204.2 mm^2^, *p* = 0.001), symptom duration (29.53 ± 44.58 months vs. 21.85 ± 34.17 months, *p* = 0.021), defect grade (62.5% IVa/IVb vs. 57.3% IVa/IVb, *p* = 0.014), integrity of corresponding joint surface (10.8% grade III–IV vs. 0.2% grade III–IV, *p* < 0.001), meniscus status (15.5% > 1/3 resected vs. 4.4% > 1/3 resected, *p* < 0.001) and number of previous surgeries (1.01 ± 1.06 vs. 0.75 ± 1.00, *p* = 0.001). In the stepwise multivariate binary logistic regression test, only the amount of varus deformity, symptom duration and quality of the corresponding joint surface remained significant predictors associated with performing a concomitant HTO.

**Conclusion:**

Based upon data from a nationwide cohort, additional HTO in context with cartilage repair procedures of the medial femoral condyle is frequently performed even in mild varus deformities less than 5°. Other factors also seem to influence decision for HTO.

## Introduction

Identification and treatment of underlying pathologies have become more recognized, concomitant surgeries more relevant in the context of cartilage surgery within recent years. Detailed preoperative patient evaluation is imperative to identify these pathologies and to enable a patient-individual treatment recommendation. Varus deformity is found in a large percentage of patients with cartilage defects of the medial femoral condyle [1, 2].

High tibial osteotomy (HTO), as the most frequently performed osteotomy around the knee, has been an established treatment option for unilateral knee osteoarthritis for decades [3–5]. Over time, concurrent cartilage treatment in patients with varus-associated osteoarthritis gained popularity, even though a benefit of an additional cartilage treatment accompanying the osteotomy compared to HTO alone could not be shown for osteoarthritic knees until now [5]. Due to innovative implants and adapted surgical techniques, the range of indications has been extended in recent years to knee joint instability, patellofemoral diseases or accompanying cartilage surgery [4, 6, 7]. In earlier years, without any scientific evidence, it seemed well accepted that correction of malalignment was indicated in patients with deformities exceeding the amount of 5°. Deformities exceeding this degree being a standard exclusion criterion for several pharmaceutical studies dealing with isolated ACI procedures indirectly suggests the same [8–11]. Interestingly, some authors demonstrated benefits of cartilage repair procedures in combination with HTO compared to cartilage repair alone even in patients with smaller deformities [12], raising the question what amount of varus deformity should be corrected. Scientific research so far has failed to show evidence supporting a 5° cut off for valgisation osteotomies of the tibia. While scientific proof of improved outcome with concomitant HTO in cartilage surgery is limited [13], it seems reasonable that orthopedic surgeons decide on performing an accompanying HTO based on the amount of leg axis malalignment.

The intention of the present study, which is based upon data from the German Cartilage Registry, was to show the current status of how cartilage surgery experts decide on performing accompanying osteotomies in regard to the degree of varus deformity.

Furthermore, aim of the present study was to analyze whether further factors influence the decision for or against HTO in the context of cartilage repair procedures or whether the decision is exclusively based on the amount of deformity. This has not been addressed in literature so far.

### Methods

Data from the German Cartilage Registry (KnorpelRegister DGOU) were evaluated for the present analysis. The KnorpelRegister DGOU is an observational, nationwide and longitudinal multi-centre registry of patients assigned for surgical treatment of cartilage defects of the knee and aims to determine real-life treatment patterns and clinical outcomes. The registry was initiated by the ‘Arbeitsgemeinschaft Klinische Geweberegeneration’ (Working Group Clinical Tissue Regeneration) of the German Society for Orthopaedics and Trauma (DGOU) in 2013. Since then, the number of sites has increased to 120. This registry is conducted in accordance with the Declaration of Helsinki and registered at germanctr.de (DRKS00005617). The current study was approved by the Ethics-Commission of the Medical Center—University of Freiburg: EK-FR 105/13_130795).

All patients aged 18 years and above that meet the following criteria are eligible to take part in the German Cartilage Registry: surgical treatment of cartilage defects of the knee, ankle or hip joint at a participating site, signed written informed consent and possession of a personal e-mail address.

Until August 2019, 4986 patients assigned for surgical treatment of cartilage defects of the knee have been included in the registry. In the present study, data from 736 patients were analyzed.

Data collection is performed using a web-based RDE System “RDE-Light” which was developed by the Clinical Trials Unit (Freiburg) as an electronic data entry interface and data management system for clinical studies and other projects in clinical research. Data are collected paperless and directly on site via an internet browser. Forms are based on HTML- and PDF-format. RDE-light is available in various languages and validated according to GAMP 5. Furthermore, it fulfils all requirements of good clinical practice (GCP). Established security standards like cryptographic security protocols (SSL/TLS), user authentication protocols and authorization concepts are applied.

After the patients sign the written informed consent, the investigator is allowed to register the patients to the database. Patient- and defect-specific parameters are reported by the treating physician at the time of surgery.

Inclusion criteria for the study at hand were existence of a leg length X-ray, a single cartilage defect on the medial femoral condyle, either no accompanying surgery or a high tibial osteotomy.

The baseline characteristic parameters degree of varus, symptom duration, corresponding joint surface status, defect size, defect stadium, age, BMI, meniscus status and the number of previous surgeries were approximately normally distributed, as assessed visually using Q–Q plots. In case of normal distribution groups were compared using a *t* test.

Univariate binary logistic regression models were used to assess the influence of baseline characteristics showing a significant difference in group comparison *t* tests on the decision to perform a concomitant HTO or not. All parameters that showed significant influence in the univariate assessment were included in a stepwise multivariate binary logistic regression model. *p* values < 0.05 were considered statistically significant. SPSS statistics version 25 was used to analyze the data.

The German Cartilage Registry is supported by a grant from the “Oscar-Helene-Stiftung” and the “Arthrosehilfe e.V.”

## Results

### Patient selection

**Fig. 1:**
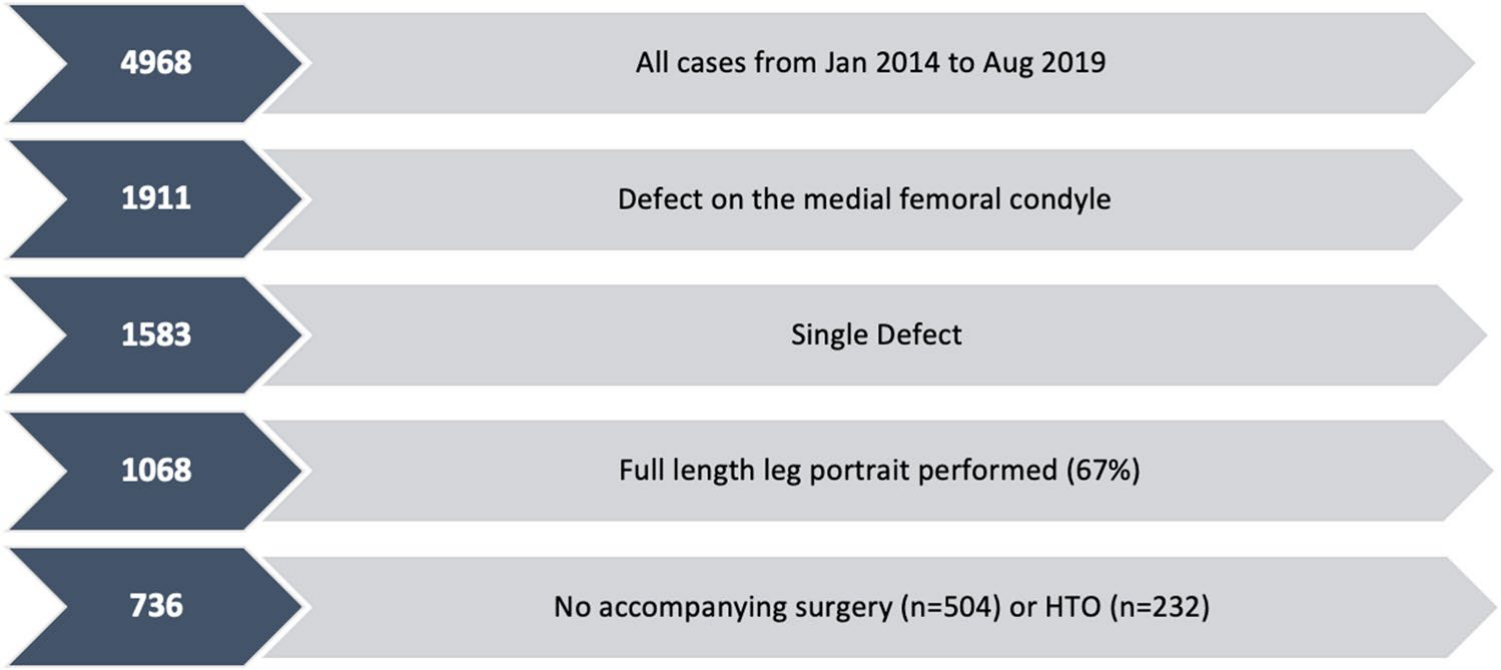
Case-inclusion matrix: out of 4968, 14.8% (*n* = 736) have been included in the analysis

From January 2014 to August 2019, 4968 datasets were entered into the German Cartilage Registry (KnorpelRegister DGOU). Only data sets of cases with a single defect on the medial femoral condyle were included. The data sets without a radiographic leg length X-ray were excluded (33%). Which means that 67% of patients undergoing cartilage surgery with a singular defect on the medial femoral condyle received an actually mandatory preoperative full-length portrait. Furthermore, only cases with either no accompanying surgery or an accompanying HTO were included (see Fig. 1).

### Demographic data

The demographic data and other baseline characteristics are shown in Table 1.

**Table 1:**
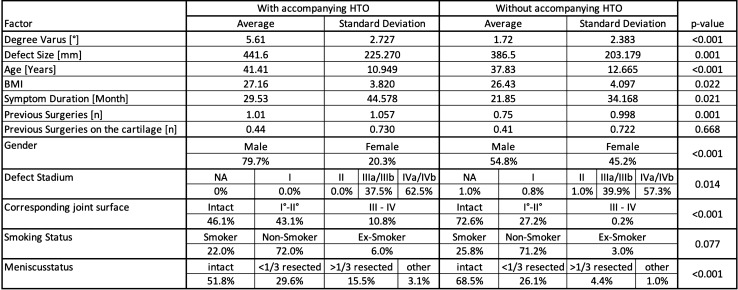
Baseline patient characteristics compared between the two groups of patients with and without concomitant HTO using *t* test to assess significant differences between the two groups

The mean age in the cohort of patients (*n* = 736) fulfilling the inclusion criteria (see Fig. 1) was 38.95 years. It was significantly higher in the group receiving an accompanying HTO (41.41 ± 10.95 years vs. 37.83 ± 12.67 years, *p* < 0.001). 62.6% (*n* = 461) of the patients were male and 37.3% (*n* = 275) were female. The quantity of female patients was significantly lower in among patients receiving a concomitant HTO with the cartilage procedure (20.3% vs. 45.2%, *p* < 0.001). The mean BMI was 26.66 and was also significantly higher in the HTO-Group (27.16 ± 3.82 vs. 26.43 ± 4.01, *p* = 0.022). 24.6% of the patients were active smokers, 2.9% were ex-smokers and 71.3% were non-smokers. There was no significant difference between both groups.

### Amount of varus, which is considered for indication of concomitant high tibial osteotomy

**Fig. 2:**
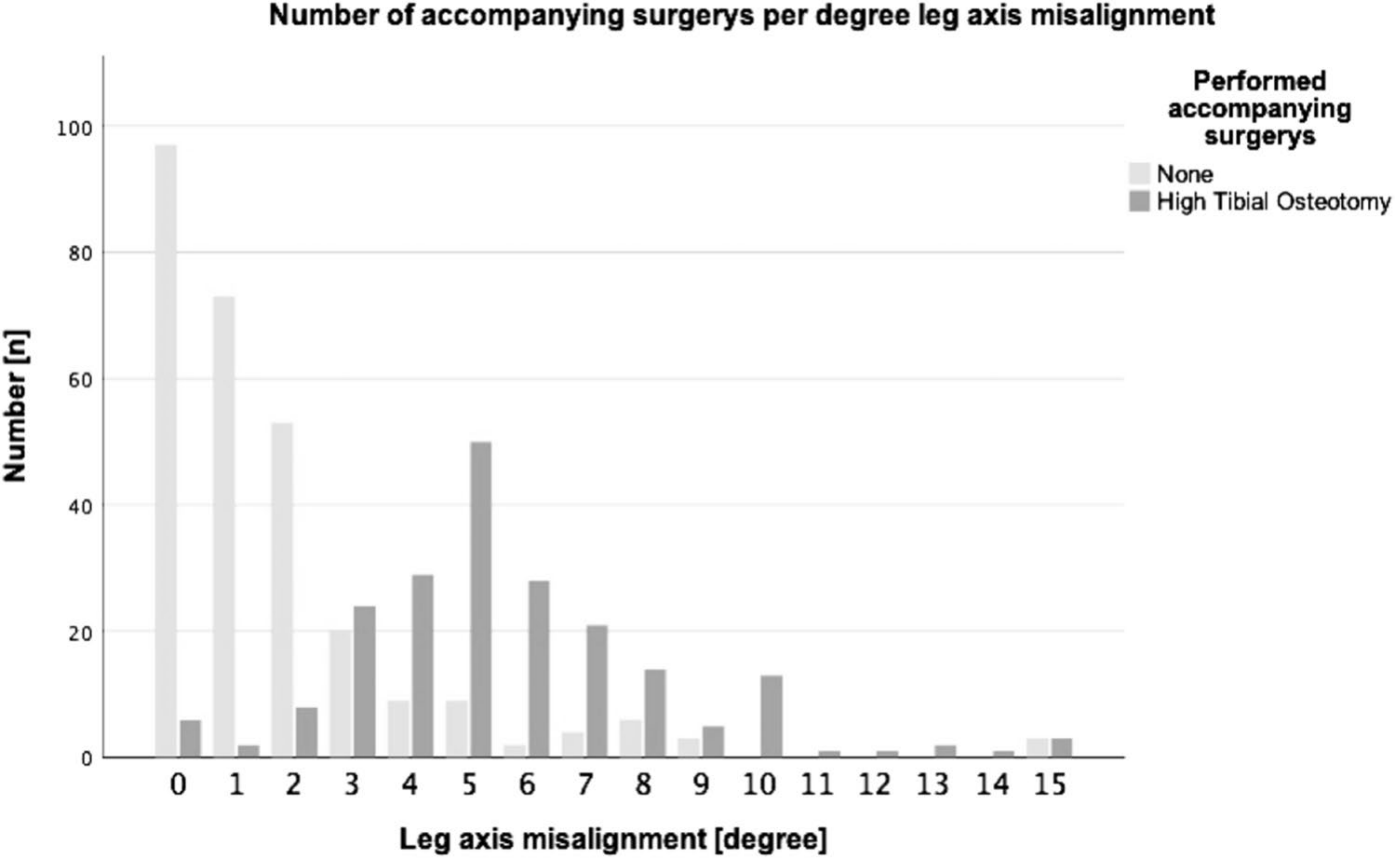
Number of accompanying surgeries per degree of leg axis malalignment. Light grey columns show the number of patients with no accompanying surgery and dark grey columns show patients with accompanying high tibial osteotomy

According to the data, accompanying HTO is sometimes performed even in a straight leg axis (see Fig. 2). The break point where more patients receive a concomitant HTO than no accompanying surgery is 3°. At 5° of malalignment and more, which is also omnipresent in literature [8–11], almost all patients receive a concomitant HTO with their cartilage procedure.

### Timing of concomitant HTO

**Fig. 3:**
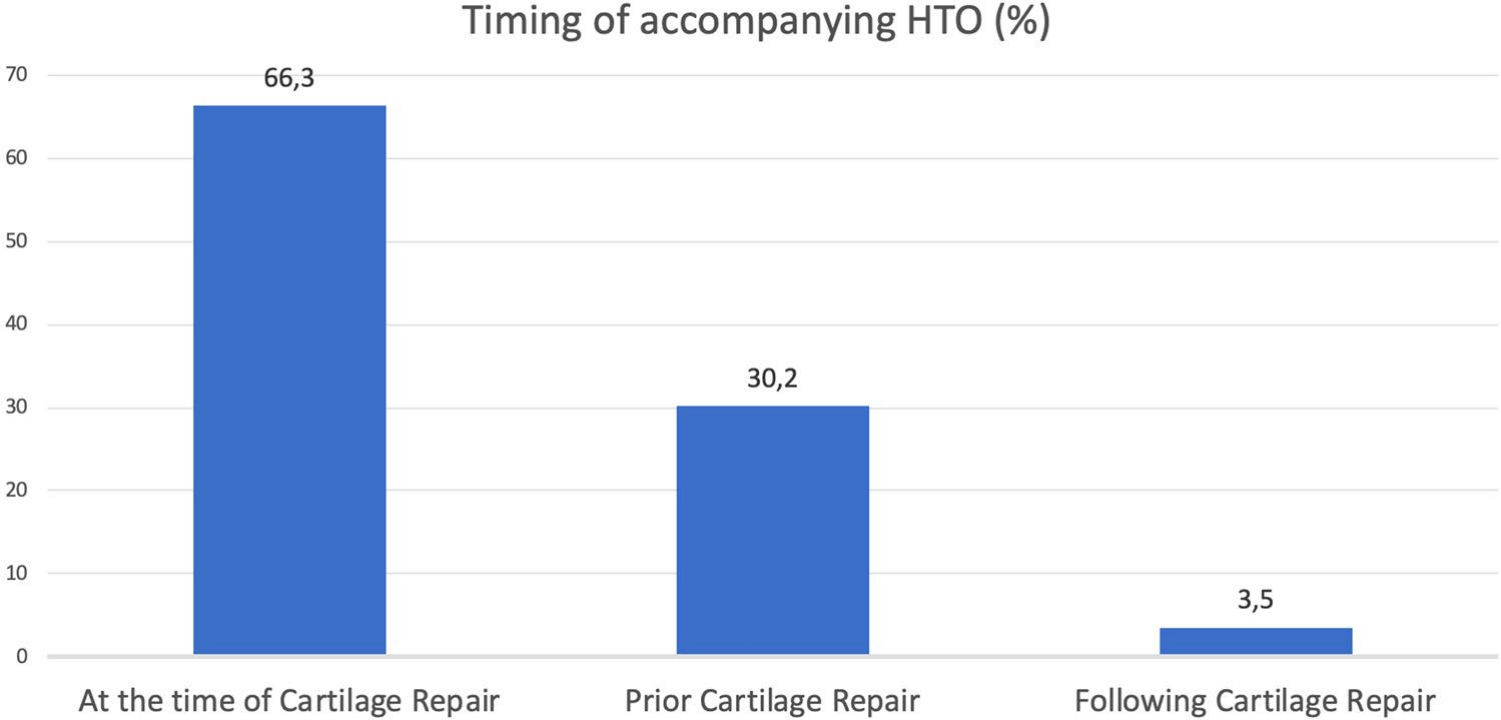
Timing of accompanying HTO in context with cartilage repair procedures

In most of the cases, the HTO was performed single session with the cartilage surgery (66.3%). In only 3.5% the HTO was performed after and in 30.2% before the cartilage procedure (see Fig. 3). Bone marrow stimulation was the most often performed procedure in a single session with the HTO (93%). ACT was performed 67% in a single session with the HTO, 29.4% before and 3.4% after the accompanying HTO.

### Parameters influencing the decision on an accompanying HTO

There were significant differences concerning age, BMI and gender between the group of patients receiving a concomitant HTO and the group of patients who did not. The degree of varus deformity not only was significantly higher in the HTO group (5.61 ± 2.73° vs. 1.72 ± 2.38°, *p* < 0.001), but also was the defect size larger (441.6 ± 225.3 mm^2^ vs, 386.5 ± 204.2 mm^2^, *p* = 0.001), the symptom duration longer (29.53 ± 44.58 months vs 21.85 ± 34.17 months, *p* = 0.021), the defect stadium higher (62.5% grade IVa/IVb vs. 57.3% grade IVa/IVb, *p* = 0.014), the corresponding joint surface more damaged (10.8% grade III–IV vs. 0.2% grade III–IV, *p* < 0.001), the meniscus status worse (15.5% > 1/3 resected vs. 4.4% > 1/3 resected, *p* < 0.001) and the number of previous surgeries significantly higher (1.01 ± 1.06 vs. 0.75 ± 1.00, *p* = 0.001). The number of previous surgeries on the cartilage and the smoking status did not differ between groups.

The amount auf varus deformity, the symptom duration, the corresponding joint surface, the defect size and stadium, as well as age, BMI, meniscus status and the number of previous surgeries were univariate predictors for a concomitant HTO.

In stepwise multivariate binary logistic regression testing, only the amount of varus deformity, the symptom duration and the quality of the corresponding joint surface remain significant predictors for a concomitant HTO with the cartilage procedure (see Table 2).

**Table 2:** Binary univariate and multivariate regression model shows all the mentioned factors as significant predictors for performing an accompanying HTO, whereas in multivariate regression, only the amount of leg axis deformity, the length of symptom duration and the quality of corresponding joint surface remain significant predictors

Factor	Univariate regressions			Multivariate regressions		
	*p* value	Exp (*B*)	CI (95%)	*p* value	Exp (*B*)	CI (95%)
Degree varus [°]	< 0.001	1.850	1.661–2.061	< 0.001	1.877	1.676–2.101
Symptom duration [months]	0.013	1.005	1.001–1.009	0.030	1.007	1.001–1.013
Corresponding joint surface*	< 0.001	3.239	2.422–4.333	0.001	2.090	1.384–3.240
Defect size [mm]	0.001	1.001	1.000–1.002			
Defect stadium**	0.028	1.363	1.033–1.799			
Age [years]	< 0.001	1.025	1.001–1.038			
BMI	0.022	1.046	1.006–1.088			
Meniscus status***	< 0.001	1.822	1.466–2.265			
Previous surgeries [*n*]	0.002	1.272	1.096–1.475			

*0 = intact, 1 = I°–II°, 2 = completely damaged

**0 = NA, 1 = I, 2 = II, 3 = IIIa/IIIB, 4 = IVa/IVb

***0 = intact, 1 ≤ 1/3 resected, 2 ≥ 1/3 resected, 3 = other

## Discussion

Concomitant surgeries aiming on the treatment of an underlying pathology have become more and more important in cartilage repair recently. This basically includes stabilization and correction of malalignment. For correction of varus deformities, HTO is well established as stand-alone therapy for progressed osteoarthritis and in combination with cartilage repair procedures in patients with focal cartilage defects of the medial compartment. Basic principle of the HTO is unloading of the medial compartment and, therefore, a neutralization of the overload caused by the varus deformity.

First important result of the present study was that in patients with cartilage defects located on the medial femoral condyle, the overall rate of concomitant high tibial osteotomy (HTO) was 46%. This is dramatically more that in the United States, where only a small number of less than 1% of patients receive an HTO in combination with cartilage resurfacing techniques [14, 15] and which is considered critically [16]. This number underlines that there is a strong belief in the efficacy of the HTO in combination with cartilage repair in Germany. The efficacy of an HTO in terms of reduction of the medial loading and clinical improvement could be demonstrated by preclinical biomechanical studies [17–19] as well as by clinical studies [20]. Also it could be shown, that even if HTO is an extra-articular procedure, it is capable to correct intra-articular varus deformity to some extend [21]. Nevertheless, indication for adjuvant leg axis correction remains not completely standardized.

While the goal of this study was primarily to analyze the everyday practice in Germany based upon a nationwide cohort of 4968 patients, it also seemed evident that many other factors would influence the decision towards performing an HTO, which have not been found in scientific literature so far.

When talking about the increasing interest in individualized medicine the identification of the underlying pathology of a cartilage defect plays a very important role. Long-leg AP radiographs have been well established to evaluate coronary alignment of the lower limb. With regard to this, the proportion of patients with femoral condyle defects being treated without prior radiographic evaluation of the lower limb alignment (33%) seems rather high—especially in the setting of a nationwide registry including centers focusing on cartilage repair procedures [22].

The role of accompanying surgeries has gained attention among surgeons during the last years and the high tibial osteotomy (HTO) for varus deformity is one of the most common concomitant treatments in cartilage surgery. Even though there is no scientific proof so far concerning the amount of deformity that can be tolerated without correction and at what amount of deformity a concomitant osteotomy should be performed, most literature recommends accompanying high tibial osteotomy when the underlying varus deformity exceeds the amount of 5°. In Germany, for reimbursement from health insurance companies, a leg axis malalignment less than 5° is even required when performing cartilage surgery [23], since those cases have been excluded from authorization and high-quality studies for autologous chondrocyte products [8–11].

Nevertheless, many experts recommend an HTO even for smaller amounts of varus deformity and a recent study demonstrated superiority of combined HTO and ACI versus ACI alone in a cohort of patients with mild varus deformity between 2° and 4° of varus [12]. These results are in line with German everyday practice, since a significant proportion of patients in the German Cartilage Registry underwent combined HTO and cartilage repair for deformities starting at 1° or 2° of varus. In our cohort accompanying HTOs were even performed in straight leg axis and the break point at which more patients received an accompanying HTO by cartilage experts was also in the range of smaller deformities (see Fig. 2). In varus malalignment greater than 3°, the majority of surgeons performed valgisation surgery in their patients. This shows that in actual clinical practice the commonly accepted edge of 5° is not present anymore, which is one of the major findings in the present study. Seemingly self-evident the data analysis showed that in the group of patients undergoing concomitant HTO, the amount of varus deformity was significantly higher (see Table 1).

Unknown until now, it became apparent, that factors other than the amount of leg axis malalignment may influence the decision towards performing a concomitant HTO: patients undergoing a concomitant HTO were not only significantly older, had a higher BMI and were more often male, they also had a longer history of symptoms, more previous surgeries of the treated knee (but not on the cartilage), a bigger defect size, a worse quality of the corresponding joint surface and a worse meniscus status which implicates that surgeons may be primarily influenced by the leg axis deformity but secondarily by all factors mentioned above. Interestingly the smoking status did not play a significant role, even though it is mentioned as a relative contraindication for HTO in literature [24]. These factors were not only highly significant in *t* testing (Table 1) but also in the univariate regression model (Table 2). In the multivariate regression, only the amount of varus deformity, the symptom duration and the quality of the corresponding joint surface remain significant predictors for performing a concomitant HTO with the cartilage procedure, since the other factors condition each other statistically. These data demonstrate that more than the amount of varus deformity seems to influence the decision for HTO in cartilage repair patients. The types of parameters associated significantly with concomitant HTO surgery suggest that more complex patients with progressed diseases and pathologies considered more complex more often receive an HTO than more “simple” cases.

On the other hand, the question could also be why not to perform a concomitant HTO in patients with an isolated cartilage defect on the medial femoral condyle. The fear of revision surgery should not be a reason, since the revision rate after concomitant HTO (2,6%) is even a little lower than the general revision rate after cartilage surgery (2,8%) [25]. Yet, a truly considerable reason which needs to be mentioned in patient consultation is the length of sick leave after high tibial osteotomies. The time of incapacity for work is 94.5 ± 77.0 days in average and even 155.0 ± 111 days in patients with heavy physical strain [26]. Furthermore, high tibial osteotomy has a relatively high complication rate which might also be a reason why surgeons avoid this concomitant surgery during cartilage repair [27, 28].

Limitations of the present study were on one hand, that the study is not fully representative since the clinics participating in the cartilage registry are hospitals with a focus on cartilage surgery, so more of an expert network than a representative subset of the German orthopedic surgeons.

This analysis brought up parameters other than the amount of varus deformity with an influence on the decision making of cartilage surgery experts towards performing a concomitant HTO. Still, it is not taken into consideration whether this influences patient outcome positively enough to compensate for patients’ preoperative characteristics such as bad meniscus status, damaged corresponding joint surface and larger defect sizes. Furthermore, the extent of correction of the leg axis has also not found any consideration. Both factors need to find recognition in further studies. Another limitation is the accuracy of the leg length X-ray which is especially error prone in small deformities due to unbalanced weight bearing or malrotation [29] and was not standardized by the operating surgeon in the underlying cohort. Nevertheless, the overall accuracy of leg alignment measurements on AP radiographs seems to have limited influence on errors in determination of the mechanical an anatomical leg axis and, therefore, should not influence the conclusions drawn by this study significantly [30].

## Conclusion

High tibial osteotomy is a well-accepted accompanying surgery in cartilage repair and is even performed in patients with a straight leg axis. Starting from 3° varus deformity more patients receive an accompanying HTO than no accompanying surgery and it is mostly performed single session with the cartilage surgery. This implicates that in a network of experts in Germany the recommendation for performing a concomitant osteotomy only in patients with deformities lager than 5° varus seems no longer tenable.

It is clear that experts see the HTO as an important concomitant treatment in cartilage surgery even for small malalignments and base their decision-making primarily upon the amount of leg axis malalignment. This is why, from the authors’ perspective, a leg length X-ray should be performed in every cartilage repair patient. This analysis also brought up additional factors contributing to the decision, not having found consideration in the literature so far. This analysis implicates that surgeons really trust on improving patient outcome with a concomitant HTO, especially in patients with more challenging preoperative findings. Yet, the efficacy of this decision, such as the influence of the individual factors on patient outcome, must be investigated in further studies to prove the clinical relevance of the findings of the present study.

## References

[1] Spahn G, Kirschbaum S, Kahl E (2006). Factors that influence high tibial osteotomy results in patients with medial gonarthritis: a score to predict the results. Osteoarthr Cartil.

[2] Spahn G, Fritz J, Albrecht D, Angele P, Fickert S, Aurich M, Hofmann GO, Niemeyer P (2017). Koinzidenz und Therapie von Beinachsdeviationen bei degenerativen Knorpelschäden des Kniegelenks. Ergebnisse aus dem Deutschen KnorpelRegister DGOU. Z Orthop Unfall.

[3] Amendola A, Panarella L (2005). High tibial osteotomy for the treatment of unicompartmental arthritis of the knee. Orthop Clin N Am.

[4] Cantin O, Magnussen RA, Corbi F, Servien E, Neyret P, Lustig S (2015). The role of high tibial osteotomy in the treatment of knee laxity: a comprehensive review. Knee Surg Sports Traumatol Arthrosc.

[5] Lee OS, Ahn S, Ahn JH, Teo SH, Lee YS (2018). Effectiveness of concurrent procedures during high tibial osteotomy for medial compartment osteoarthritis: a systematic review and meta-analysis. Arch Orthop Trauma Surg.

[6] Savarese E, Bisicchia S, Romeo R, Amendola A (2011). Role of high tibial osteotomy in chronic injuries of posterior cruciate ligament and posterolateral corner. J Orthop Traumatol.

[7] Niemeyer P, Kreuz PC, Steinwachs M, Südkamp NP (2007). Chirurgische Therapieverfahren zur Behandlung Umschriebener Knorpelschäden am Kniegelenk. Sportverletz Sportschaden.

[8] Niemeyer P, Albrecht D, Andereya S, Angele P, Ateschrang A, Aurich M, Baumann M, Bosch U, Erggelet C, Fickert S, Gebhard H, Gelse K, Günther D, Hoburg A, Kasten P, Kolombe T, Madry H, Marlovits S, Meenen NM, Müller PE, Nöth U, Petersen JP, Pietschmann M, Richter W, Rolauffs B, Rhunau K, Schewe B, Steinert A, Steinwachs MR, Welsch GH, Zinser W, Fritz J (2016). Autologous chondrocyte implantation (ACI) for cartilage defects of the knee: a guideline by the working group “Clinical Tissue Regeneration” of the German Society of Orthopaedics and Trauma (DGOU). Knee.

[9] Niemeyer P, Laute V, Zinser W, Becher C, Kolombe T, Fay J, Pietsch S, Kuźma T, Widuchowski W, Fickert S (2019). A prospective, randomized, open-label, multicenter, phase III noninferiority trial to compare the clinical efficacy of matrix-associated autologous chondrocyte implantation with spheroid technology versus arthroscopic microfracture for cartilage defects of the knee. Orthop J Sport Med.

[10] Saris D, Price A, Widuchowski W, Bertrand-Marchand M, Caron J, Drogset JO, Emans P, Podskubka A, Tsuchida A, Kili S, Levine D, Brittberg M (2014). Matrix-applied characterized autologous cultured chondrocytes versus microfracture: two-year follow-up of a prospective randomized trial. Am J Sports Med.

[11] Brittberg M, Recker D, Ilgenfritz J, Saris DBF (2018) Matrix-applied characterized autologous cultured chondrocytes versus microfracture: five-year follow-up of a prospective randomized trial. Am J Sports Med 46(6):1343–1351. https://www.ncbi.nlm.nih.gov/pubmed/29565642 10.1177/0363546518756976 29565642

[12] Bode G, Schmal H, Pestka JM, Ogon P, Südkamp NP, Niemeyer P (2013). A non-randomized controlled clinical trial on autologous chondrocyte implantation (ACI) in cartilage defects of the medial femoral condyle with or without high tibial osteotomy in patients with varus deformity of less than 5. Arch Orthop Trauma Surg.

[13] Kahlenberg CA, Nwachukwu BU, Hamid KS, Steinhaus ME, Williams RJ (2017). Analysis of outcomes for high tibial osteotomies performed with cartilage restoration techniques. Arthrosc J Arthrosc Relat Surg.

[14] Hancock KJ, Westermann RR, Shamrock AG, Duchman KR, Wolf BR, Amendola A (2019). Trends in knee articular cartilage treatments: an american board of orthopaedic surgery database study. J Knee Surg.

[15] Frank RM, Cotter EJ, Hannon CP, Harrast JJ, Cole BJ (2019). Cartilage restoration surgery: incidence rates, complications, and trends as reported by the American Board of Orthopaedic Surgery Part II candidates. Arthrosc J Arthrosc Relat Surg.

[16] Westermann RW (2019). Editorial commentary: when performing cartilage restoration, please don’t put down the osteotomy saw!. Arthrosc J Arthrosc Relat Surg.

[17] Mina C, Garrett WE, Pietrobon R, Glisson R, Higgins L (2008). High tibial osteotomy for unloading osteochondral defects in the medial compartment of the knee. Am J Sports Med.

[18] Agneskirchner JD, Hurschler C, Wrann CD, Lobenhoffer P (2007). The effects of valgus medial opening wedge high tibial osteotomy on articular cartilage pressure of the knee: a biomechanical study. Arthrosc J Arthrosc Relat Surg.

[19] Agneskirchner JD, Hurschler C, Stukenborg-Colsman C, Imhoff AB, Lobenhoffer P (2004). Effect of high tibial flexion osteotomy on cartilage pressure and joint kinematics: a biomechanical study in human cadaveric knees. Winner of the AGA-DonJoy Award 2004. Arch Orthop Trauma Surg.

[20] Floerkemeier S, Staubli AE, Schroeter S, Goldhahn S, Lobenhoffer P (2013). Outcome after high tibial open-wedge osteotomy: a retrospective evaluation of 533 patients. Knee Surg Sport Traumatol Arthrosc.

[21] Ji W, Luo C, Zhan Y, Xie X, He Q, Zhang B (2019). A residual intra-articular varus after medial opening wedge high tibial osteotomy (HTO) for varus osteoarthritis of the knee. Arch Orthop Trauma Surg.

[22] Hinman RS, May RL, Crossley KM (2006). Is there an alternative to the full-leg radiograph for determining knee joint alignment in osteoarthritis?. Arthritis Care Res.

[23] Gemeinsamer Bundesausschuss (2009) Matrixassoziierte autologe Chondrozytenimplantation am Kniegelenk [Internet]. https://www.g-ba.de/downloads/40-268-942/2009-07-17-MACI-Knie.pdf. Accessed 9 Nov 2019

[24] Niemeyer P, Stöhr A, Köhne M, Hochrein A (2017). Medial opening wedge high tibial osteotomy. Oper Orthop Traumatol.

[25] Pestka JM, Luu NH, Südkamp NP, Angele P, Spahn G, Zinser W, Niemeyer P (2018). Revision surgery after cartilage repair: data from the german cartilage registry (KnorpelRegister DGOU). Orthop J Sport Med.

[26] Bode G, Ogon P, Pestka J, Zwingmann J, Feucht M, Südkamp N, Niemeyer P (2015). Clinical outcome and return to work following single-stage combined autologous chondrocyte implantation and high tibial osteotomy. Int Orthop.

[27] Attinger MC, Behrend H, Jost B (2014). Complete rupture of the popliteal artery complicating high tibial osteotomy. J Orthop.

[28] Woodacre T, Ricketts M, Evans JT, Pavlou G, Schranz P, Hockings M, Toms A (2016) Complications associated with opening wedge high tibial osteotomy - a review of the literature and of 15 years of experience. Knee 23(2):276–282. https://www.ncbi.nlm.nih.gov/pubmed/26596554 10.1016/j.knee.2015.09.018 26596554

[29] Jakma TSC, Verhaar JAN, Brouwer KH, Brouwer RW (2007). Pitfalls in determining knee alignment. J Knee Surg.

[30] Khare R, Jaramaz B (2017). Accuracy of leg alignment measurements from antero-posterior radiographs. Biomed Tech.

