# Correction to: Decision making for concomitant high tibial osteotomy (HTO) in cartilage repair patients based on a nationwide cohort study of 4968 patients

**DOI:** 10.1007/s00402-021-04014-8

**Published:** 2021-07-13

**Authors:** Svea Faber, Johannes Zellner, Peter Angele, Gunter Spahn, Ingo Löer, Wolfgang Zinser, Philipp Niemeyer

**Affiliations:** 1 OCM | Orthopädische Chirurgie München, Steinerstrasse 6, 812306 München, Germany; 2 St. Joseph Krankenhaus, Regensburg, Germany; 3 Sporthopaedicum, Berlin, Straubing, Regensburg, Germany; 4 Klinik für Unfallchirurgie, Universitätsklinikum Regensburg, Regensburg, Germany; 5 Praxisklinik Eisenach, Eisenach, Germany; 6 Klinik für Unfall-, Hand- und Wiederherstellungschirurgie, Universitätsklinikum Jena, Jena, Germany; 7 Orthopädie in Essen, Essen, Germany; 8 St. Vinzenz-Hospital, Dinslaken, Germany; 9 Klinik für Orthopädie und Traumatologie, Universtitätsklinikum Freiburg, Freiburg, Germany

## Correction to: Archives of Orthopaedic and Trauma Surgery (2020) 140:1437–1444 10.1007/s00402-020-03476-6

The article Decision making for concomitant high tibial osteotomy (HTO) in cartilage repair patients based on a nationwide cohort study of 4968 patients, written by Svea Faber, Johannes Zellner, Peter Angele, Gunter Spahn, Ingo Löer, Wolfgang Zinser and Philipp Niemeyer was originally published Online First without Open Access. After publication in volume 140, issue 10, page 1437–1444 the author decided to opt for Open Choice and to make the article an Open Access publication. Therefore, the copyright of the article has been changed to © The Author(s) 2021 and This article is licensed under a Creative Commons Attribution 4.0 International License, which permits use, sharing, adaptation, distribution and reproduction in any medium or format, as long as you give appropriate credit to the original author(s) and the source, provide a link to the Creative Commons licence, and indicate if changes were made. The images or other third party material in this article are included in the article’s Creative Commons licence, unless indicated otherwise in a credit line to the material. If material is not included in the article’s Creative Commons licence and your intended use is not permitted by statutory regulation or exceeds the permitted use, you will need to obtain permission directly from the copyright holder. To view a copy of this licence, visit http://creativecommons.org/licenses/by/4.0/.

The original article has been corrected.

Open Access funding enabled and organized by Projekt DEAL.

